# Charting the course of depression care: a meta-analysis of reliability generalization of the patient health questionnaire (PHQ- 9) as the measure

**DOI:** 10.1007/s44192-025-00181-x

**Published:** 2025-04-07

**Authors:** Kenni Wojujutari Ajele, Erhabor Sunday Idemudia

**Affiliations:** Faculty of Humanities, North-West University, Mafikeng, South Africa

**Keywords:** Depression, PHQ- 9, Reliability generalization, Meta-analysis, Cultural adaptation

## Abstract

**Background:**

Depression is a leading cause of disability worldwide, requiring reliable and adaptable screening tools. The Patient Health Questionnaire- 9 (PHQ- 9) is widely used, yet its reliability across diverse populations and cultural adaptations remains unclear.

**Objective:**

This meta-analysis assessed the reliability generalization of the PHQ- 9 across different populations, settings, and cultural contexts to determine its applicability in global mental health assessments.

**Methods:**

A reliability generalization (RG) meta-analysis was conducted on 60 studies with 232,147 participants. A random-effects model was used to estimate pooled internal consistency (Cronbach’s α) and test–retest reliability. Subgroup analyses examined the effects of administration modes, cultural adaptations, and study settings.

**Results:**

The pooled Cronbach’s α was 0.86 (95% CI [0.85, 0.87]), indicating high internal consistency. Test–retest reliability, based on eight studies, was 0.82 (95% CI [0.74, 0.90]). Self-administered formats had the highest reliability (α = 0.87), while face-to-face interviews were lower (α = 0.80). Substantial heterogeneity (I^2^ = 99.3%) was observed.

**Conclusions:**

The PHQ- 9 is a reliable depression screening tool globally, but significant heterogeneity highlights the need for continued cultural adaptation and validation to enhance its applicability across diverse contexts.

## Introduction

Depression is a leading cause of disability worldwide, requiring effective and reliable screening tools for early detection and intervention [1, 2]. The Patient Health Questionnaire (PHQ- 9) is one of the most widely used tools for assessing depression severity due to its alignment with DSM-IV criteria, ease of use, and clinical utility [3]. It consists of nine items assessing symptom frequency over the past two weeks, allowing clinicians to determine depression severity and treatment needs.

Despite its widespread application, concerns remain about the PHQ- 9’s reliability across different populations, languages, administration methods, and clinical settings [4, 5]. While studies in the U.S., Peru, and Germany have found consistent internal consistency values [3, 6], research has also highlighted differences in factorial structure and dimensionality across cultural contexts [7, 8]. This variability raises critical concerns about whether the PHQ- 9 is equally reliable across diverse populations and whether moderators such as administration mode (self-report vs. interview) or cultural adaptations affect its reliability.

The PHQ- 9’s internal consistency has been widely studied, with Cronbach’s alpha consistently reported as high [9–11]. However, test–retest reliability, which measures the PHQ- 9’s stability over time, has yielded mixed results across settings and populations. Studies have found acceptable test–retest reliability among Filipino migrant workers [12], psychiatric patients [13, 14], and digital versus paper-based assessments [15]. While these studies provide valuable insights, they are often conducted in isolation, limiting the ability to draw generalizable conclusions about the PHQ- 9’s stability and consistency across different conditions.

Furthermore, adaptations of the PHQ- 9 for specific cultural groups have demonstrated its applicability in Ethiopia [9], Mozambique [16], Vietnam [17], Somalia [18], and Quechua-speaking populations in Peru [19, 20]. However, the reliability of these adaptations varies, further underscoring the need for a systematic evaluation. Given these inconsistencies, a meta-analysis using reliability generalization** (**RG) is needed. RG is a statistical method that evaluates the variability of a measure’s reliability across studies, populations, and contexts, helping identify factors that contribute to reliability differences [21, 22].

This study aims to conduct an RG meta-analysis to systematically evaluate PHQ- 9 reliability across diverse populations, settings, and administration methods. This study seeks to determine the overall reliability (Cronbach’s alpha and test–retest reliability) of the PHQ- 9 across diverse populations and settings, examine how reliability varies across cultural adaptations, administration methods, and clinical versus general populations, and identify factors contributing to heterogeneity in PHQ- 9 reliability across studies. By systematically analysing existing data, this RG provides a comprehensive evaluation of the PHQ- 9’s reliability across global contexts. The findings will inform future research and clinical practice by identifying key factors that influence PHQ- 9 reliability, ensuring its appropriate use in depression screening worldwide.

## Methods

### Design

We conducted a reliability generalization (RG) meta-analysis to evaluate the psychometric properties of the Patient Health Questionnaire- 9 (PHQ- 9) across diverse populations and settings. The RG approach systematically examines variations in reliability estimates across studies, identifying factors that influence measurement consistency. A random-effects model was used to account for expected differences between studies, ensuring that findings reflect a broad range of populations, administration methods, and cultural adaptations. A pooled estimate does not imply that reliability is fixed across populations but rather serves as a benchmark while accounting for heterogeneity. This approach allows for the identification of moderators that may contribute to variability in PHQ- 9 reliability, guiding its appropriate application in different contexts.

To enhance transparency and methodological rigor, we pre-registered our study protocol in PROSPERO (CRD42024508766) and adhered to PRISMA guidelines [23] for study selection, data extraction, and analysis. These methodological safeguards minimize bias and enhance the reproducibility of findings.

### Search strategy

The meta-analysis on the reliability generalisation of the Patient Health Questionnaire (PHQ- 9) employed a detailed search strategy across significant databases, including PsycINFO, Web of Science, Scopus, and Google Scholar. The time frame for the search extended from January 1, 2015, to the current date, focusing exclusively on English-language, peer-reviewed journal articles. To identify the most pertinent studies, we employed a combination of keywords such as “Patient Health Questionnaire- 9” and “PHQ- 9”. “Patient Health Questionnaire”, “reliability”, “psychometrics properties”, “internal consistency”, and “test–retest reliability”. Additionally, The combining keywords with Boolean operators for the search strategy such as (“Patient Health Questionnaire- 9” OR “Patient Health Questionnaire” OR “PHQ- 9”) AND (“reliability” OR “internal consistency” OR “test–retest reliability” OR “psychometrics properties”). This approach ensures a comprehensive review of the literature on the PHQ- 9’s reliability across different populations, settings, and study designs, including published and grey literature. Manual searches of reference lists and direct author inquiries supplemented database searches to identify additional relevant studies.

### Inclusion and exclusion criteria

This review follows clear inclusion and exclusion criteria to analyse the reliability of the Patient Health Questionnaire (PHQ- 9) across diverse populations and settings. Studies were included if they assessed PHQ- 9 reliability in adolescents to the elderly, ensuring lifespan applicability. We considered research from varied socio-cultural and geographical backgrounds, reinforcing cross-cultural validity. Both clinical and non-clinical populations were included to evaluate PHQ- 9 reliability across mental health settings. Studies assessing all gender identities were considered to ensure broad applicability. We included research utilizing the original English version and validated translations, provided they reported reliability metrics such as Cronbach’s alpha, test–retest reliability, or inter-rater reliability.

We excluded studies focusing on populations outside adolescence to old age, those lacking cultural or geographic diversity, and research examining only clinical or non-clinical groups without comparative analysis. Studies omitting gender diversity, using unvalidated PHQ- 9 versions, or failing to report key reliability metrics were also excluded. This systematic approach ensures a comprehensive evaluation of PHQ- 9’s reliability, offering insights into its applicability, adaptability, and consistency across different contexts and populations.

### Quality assessment

We conducted a comprehensive quality assessment using the COSMIN Checklist [24], designed for evaluating patient-reported outcome measures, and adapted relevant domains from QUADAS- 2 [25] to assess the reliability of diagnostic tools. This evaluation examined sample selection, study design, administration methods, and statistical techniques to ensure methodological rigor.

Key areas assessed included population representativeness, adequacy of sample sizes, appropriateness of PHQ- 9 administration methods, and clarity in reporting reliability findings. Statistical methods used to calculate Cronbach’s alpha, test–retest reliability, and inter-rater reliability were scrutinized for robustness and transparency. Two independent reviewers conducted the assessments, resolving disagreements through discussion or consultation with a third reviewer. This dual-review process, combined with established assessment tools, ensured a systematic, unbiased, and rigorous evaluation of study quality, forming a robust foundation for the meta-analysis conclusions.

### Data extraction and study selection

We employed a systematic approach to data extraction and study selection using Covidence (https://app.covidence.org) and Excel to assess the reliability generalization of the Patient Health Questionnaire (PHQ- 9). Data extraction was conducted using a pre-designed form, capturing study characteristics, PHQ- 9 assessment details, reliability measures (Cronbach’s α, test–retest reliability), language adaptations, administration mode, sample demographics, and study settings. The form was pilot tested on a subset of studies to ensure accuracy and consistency.

Two independent reviewers used Covidence for study screening, selection, and data extraction, minimizing bias and ensuring transparency. Extracted data were then systematically recorded and analysed in Excel. Discrepancies were resolved through consensus or third-party adjudication. For study selection, titles and abstracts were screened in Covidence against predefined inclusion and exclusion criteria by two reviewers. Eligible studies underwent full-text review, with reasons for exclusions documented. Only studies meeting all criteria were included in the final review, ensuring a transparent, standardized, and reproducible evaluation process.

### Statistical analysis

The statistical analysis was performed using RStudio 2024.12.0 + 467, utilizing the “meta” package for meta-analytic computations and the “readxl” package for importing data from Excel files. The “meta” package was selected for its comprehensive functionality in conducting random-effects meta-analyses, subgroup analyses, heterogeneity assessments, and visualizing results through forest and funnel plots. It provides robust methods for estimating pooled effect sizes and handling between-study variability, making it well-suited for reliability generalization meta-analysis. A random effects model was employed to calculate pooled estimates of Cronbach’s α and 95% confidence intervals (CI), accounting for between-study variability. Subgroup analyses were conducted to explore potential moderators, such as region, language, mode of administration, setting, and sample size. Subgroups were categorized using the “byvar” argument within the meta package, which enables stratified analyses and the generation of subgroup-specific forest plots. Overall and subgroup-specific estimates were visualized in forest plots.

Heterogeneity was assessed using the Q statistic, its corresponding p-value, and the I^2^ statistic, which quantifies the proportion of variability attributable to heterogeneity rather than chance. The between-study variance was estimated using τ^2^ (tau-squared). Differences across subgroups were tested using the test for subgroup differences, reported as the Q statistic and p-value. Potential publication bias was examined through visual inspection of funnel plots. Publication bias was assessed through funnel plots. All statistical significance was set at p < 0.05. The combination of “meta” and “readxl” packages streamlined data handling and analysis.

## Results

### PRISMA flow diagram

**Fig. 1:**
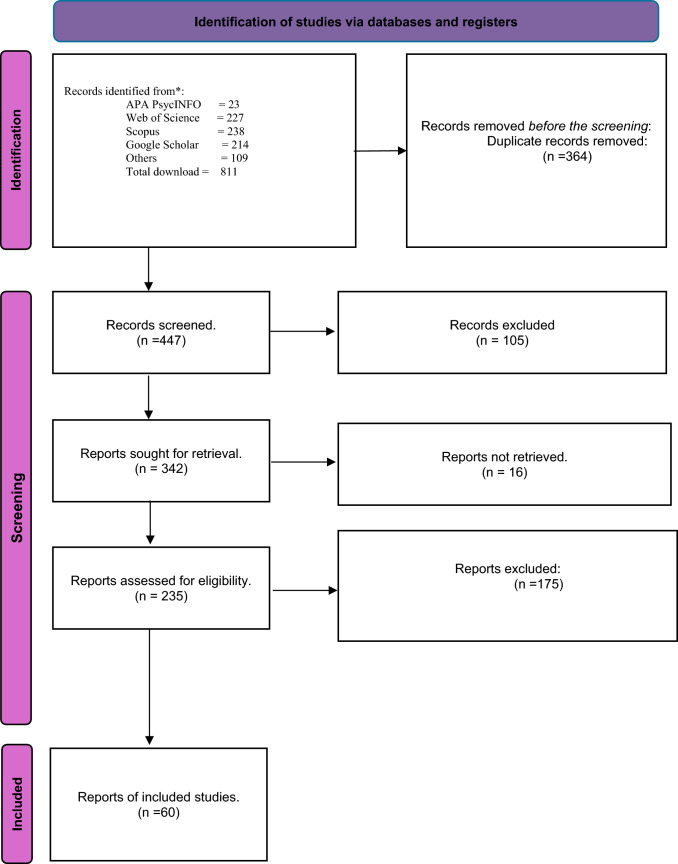
PRISMA 2020 flow diagram of the studies selection process

The study selection process is summarized in the PRISMA 2020 flow diagram (Fig. 1). A total of 811 records were identified from various sources. After the removal of 364 duplicate records, 447 unique records were screened. Of these, 105 records were excluded during the title and abstract screening process. Following this, 342 reports were sought for retrieval, and 16 could not be retrieved. A total of 235 reports were assessed for eligibility, with 175 excluded based on the predefined inclusion and exclusion criteria. Finally, 60 studies met the eligibility criteria and were included in the meta-analysis. The detailed steps of the study selection process are visually represented in Fig. 1, ensuring transparency and reproducibility of the methodology. This selection process followed the PRISMA guidelines [23].

### Characteristics of the included studies

The total sample size across all studies was diverse, ranging from 47 participants [15, 26] to 90,846 participants [27]. Studies were conducted across multiple regions, including Asia [4, 9, 19, 28–34], Africa [16, 35–40], Europe [10, 13, 41–44], North America [4, 13, 45, 46], and South America [47–50]. Mixed regions were also represented in large-scale studies [5]. Cronbach’s α values, representing internal consistency reliability, ranged from 0.66 [15]to 0.95 [34], with some studies reporting test–retest reliability [28, 41]. Various adaptations of the PHQ- 9 were used, including English [4, 28, 45], Spanish [42, 48], Chinese [29, 31, 51], and Quechua [20, 34]. Settings spanned primary healthcare [4, 10, 48], general population studies [50, 52], psychiatric inpatients [4, 6, 34], and educational institutions [32, 33]. Most studies employed self-administered modes of administration, though interviews and face-to-face methods were also reported [16, 30, 39]. The included studies highlight the global applicability and adaptability of the PHQ- 9 across diverse populations, languages, and settings.

### Reliability of the PHQ- 9: a meta-analysis

**Fig. 2:**
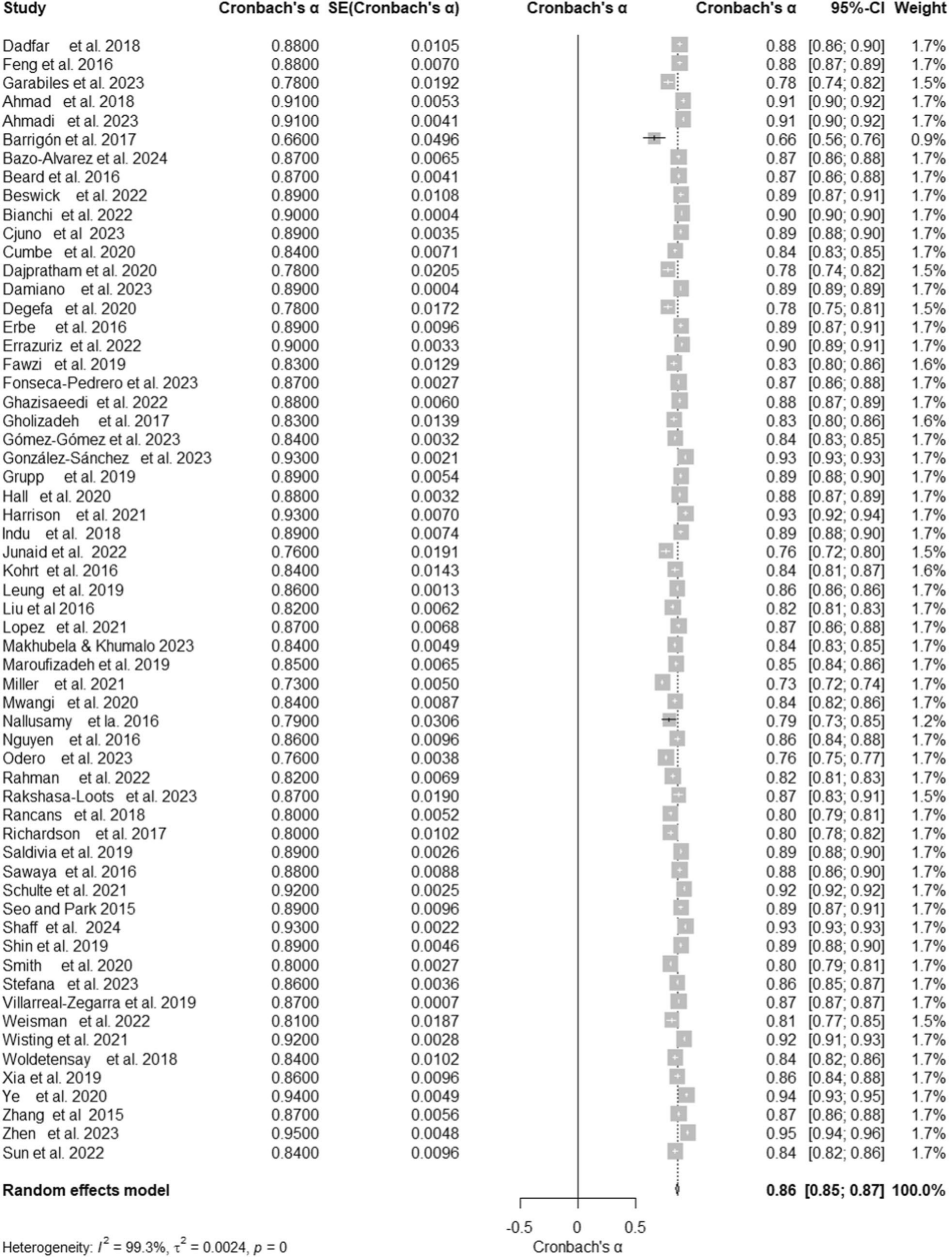
Forest Plot of Cronbach’s α Reliability Estimates for the PHQ- 9 Across Studies. Forest plot showing Cronbach’s α reliability estimates for the PHQ- 9 across studies. The pooled estimate from the random-effects model is displayed alongside individual study weights and confidence intervals

**Fig. 3:**
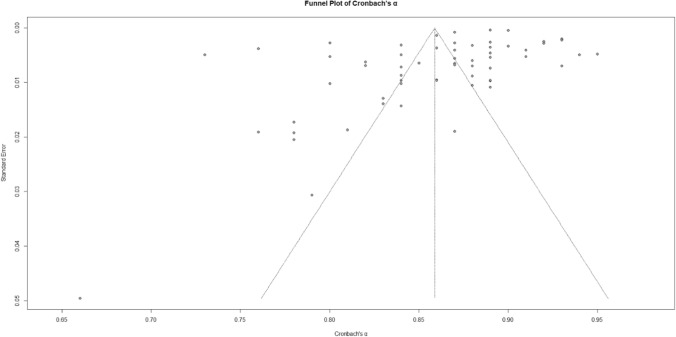
Funnel Plot of Cronbach’s α for the PHQ- 9 Across Studies. The funnel plot illustrates the distribution of Cronbach’s α estimates for the PHQ- 9 against the standard error. Symmetry around the pooled estimate indicates potential publication bias or heterogeneity among included studies

A meta-analysis of 60 studies, with a total sample size of 232,147, evaluated the reliability of the Patient Health Questionnaire- 9 (PHQ- 9). The analysis yielded an overall Cronbach’s α of 0.86 (95% CI [0.85, 0.87]), as shown in the forest plot (Fig. 2), indicating high internal consistency across studies. Substantial heterogeneity was observed, with I^2^ = 99.3%, τ^2^ = 0.0024, and a significant test of heterogeneity, Q (59) = 7981.54, p < 0.001 (Table 1). The funnel plot (Fig. 3) revealed some asymmetry, suggesting potential publication bias or heterogeneity. Despite this, the high reliability estimate supports the robustness of the PHQ- 9 as a reliable assessment tool. The z-test confirmed the significance of this estimate, z = 133.20, p < 0.001.

**Table 1:** Summary of Pooled Reliability Estimates (Cronbach’s α) for the PHQ- 9 Across Subgroups

Total/subgroup	K	Cronbach’s α	z	95%–CI	Heterogeneity		
					Q	I^2^	τ^2^
PHQ- 9 (overall reliability)	60				7981.54**	99.3%	0.0024
Random effects model		0.86	133.20**	0.85; 0.87			
Test–retest	8				667.07**	99.0%	0.0126
Random effects model		0.82	20.26**	0.74; 0.90			
Languages							
Others	23	0.86		0.84; 0.87	637.80	96.6%	0.0016
Chinese	7	0.87		0.84; 0.90	309.36	98.1%	0.0014
English	12	0.87		0.84; 0.89	728.00	98.5%	0.0025
Spanish	8	0.85		0.81; 0.90	1742.04	99.6%	0.0042
Quechua	3	0.90		0.86; 0.95	135.90	98.5%	0.0017
More than one	5	0.82		0.75; 0.88	2815.91	99.9%	0.0058
Portuguese	2	0.86		0.82; 0.91	48.90	98.0%	0.0012
Settings							
Educational	7	0.84		0.81; 0.88	94.15	93.6%	0.0020
Primary healthcare	24	0.85		0.83; 0.87	3162.38	99.3%	0.0023
General	18	0.86		0.84; 0.88	3257.80	99.5%	0.0025
Mental health	8	0.88		0.86; 0.91	276.93	97.5%	0.0017
Rehabilitation	3	0.87		0.78; 0.96	46.18	95.7%	0.0061
Mode of administration							
Self-administered	47	0.87		0.86; 0.88	4207.55	98.9%	0.0016
Interview-based	11	0.81		0.78; 0.84	3347.41	99.7%	0.0023
Face-to-face	2	0.80		0.72; 0.88	17.09	94.1%	0.0030
Region							
Global South	41	0.85		0.84; 0.87	5387.49	99.3%	0.0023
Global North	17	0.87		0.84; 0.89	1448.94	94 98.9%	0.0027
Other	2	0.86		0.77; 0.94	23.10	10 95.7%	0.0039
Sample size							
Small sample (≤ 500)	33	0.86		0.84; 0.87	792.78	96.0%	0.0025
Large sample (> 500)	27	0.86		0.84; 0.88	7188.31	99.6%	0.0024

95% *CI* Confidence interval, *Q* Cochran’s Q statistic for heterogeneity

k = Number of studies in each subgroup

Cronbach’s α = Internal consistency reliability estimate

z = Test statistic for overall effect size

I^2^ = Percentage of total variability due to heterogeneity

τ^2^ = Between-study variance estimate

^**^Significance: p < 0.05

### Test–retest reliability of the PHQ- 9: a meta-analysis

**Fig. 4:**
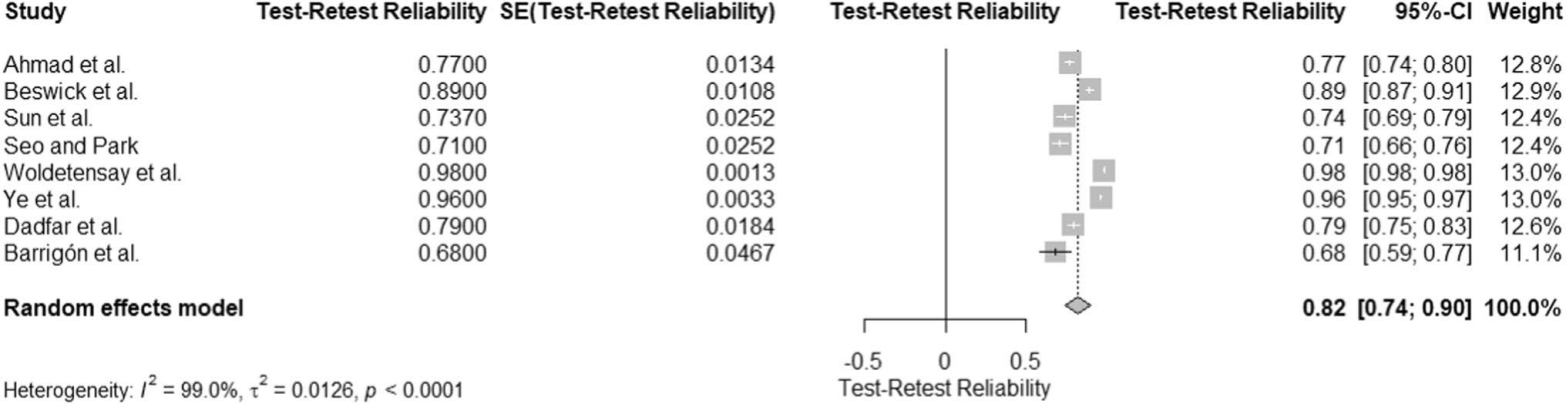
Forest Plot of Test–Retest Reliability Estimates for the PHQ- 9 Across Studies. This forest plot shows test–retest reliability estimates for the PHQ- 9 across studies. Individual study estimates, confidence intervals, and weights are displayed. Substantial heterogeneity is observed

A meta-analysis of 8 studies, with a total sample size of 1,208, evaluated the test–retest reliability of the Patient Health Questionnaire- 9 (PHQ- 9). The analysis yielded an overall test–retest reliability estimate of 0.82 (95% CI [0.74, 0.90]), as shown in the forest plot (Fig. 4), indicating strong reliability across repeated administrations. Substantial heterogeneity was observed, with I^2^ = 99.0%, τ^2^ = 0.0126, and a significant test of heterogeneity, Q (7) = 667.07, p < 0.0001. The high heterogeneity (I^2^ = 99.0%) suggests that variability among test–retest reliability estimates could stem from methodological differences or sample characteristics. Despite this, the overall reliability estimate supports the PHQ- 9 as a consistent tool for assessing depressive symptoms over time. The z-test confirmed the significance of the reliability estimate, z = 20.26, p < 0.0001 (Table 1).

### PHQ- 9 reliability across languages

**Fig. 5:**
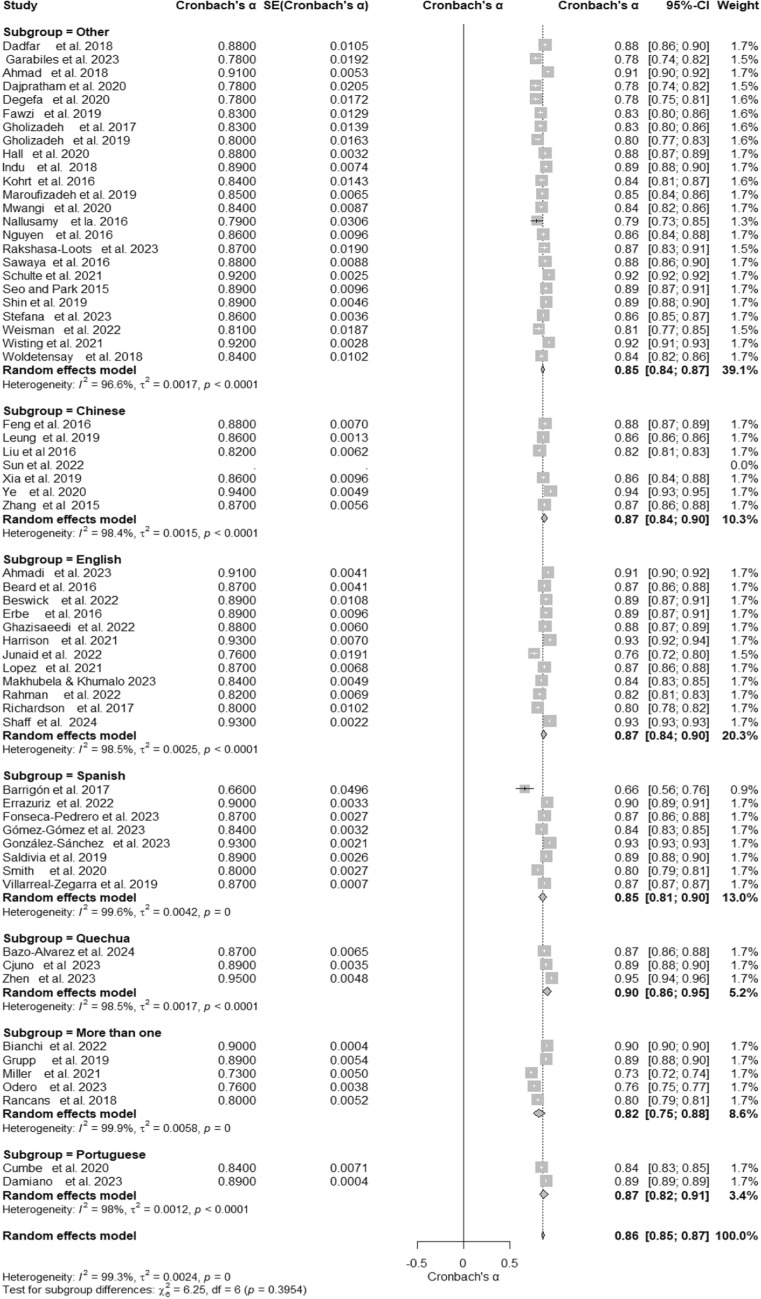
Subgroup Forest Plot of Cronbach’s α for the PHQ- 9 by Language. This forest plot presents Cronbach’s α reliability estimates for the PHQ- 9, stratified by language subgroups (e.g., Chinese, English, Spanish). Individual study estimates, subgroup pooled estimates, and their corresponding confidence intervals are shown. Heterogeneity is assessed within and across subgroups

A meta-analysis of 60 studies, including various language adaptations of the PHQ- 9, evaluated its internal consistency reliability. The overall Cronbach’s α was 0.86 (95% CI [0.85, 0.87]), as displayed in the forest plot (Fig. 5) and detailed in the summary table (Table 1), reflecting high reliability across diverse samples. Heterogeneity was substantial, with I^2^ = 99.3%, τ^2^ = 0.0024, and a significant test of heterogeneity, Q (59) = 7981.54, p < 0.001. Subgroup analyses revealed similar reliability levels across different languages. Subgroups for Chinese (α = 0.87, 95% CI [0.84, 0.90]), English (α = 0.87, 95% CI [0.84, 0.90]), Spanish (α = 0.85, 95% CI [0.81, 0.90]), and Portuguese (α = 0.87, 95% CI [0.82, 0.91]) demonstrated high internal consistency. The Quechua subgroup reported the highest reliability (α = 0.90, 95% CI [0.86, 0.95]), while the “More than one” subgroup had the lowest reliability (α = 0.82, 95% CI [0.75, 0.88]). Although heterogeneity was high across subgroups, as shown in Table 1 and the forest plot (Fig. 5), the test for subgroup differences was not significant, Q (6) = 5.62, p = 0.4671. This suggests consistency in Cronbach’s α across languages despite methodological and sample differences.

### PHQ- 9 reliability across different settings

**Fig. 6:**
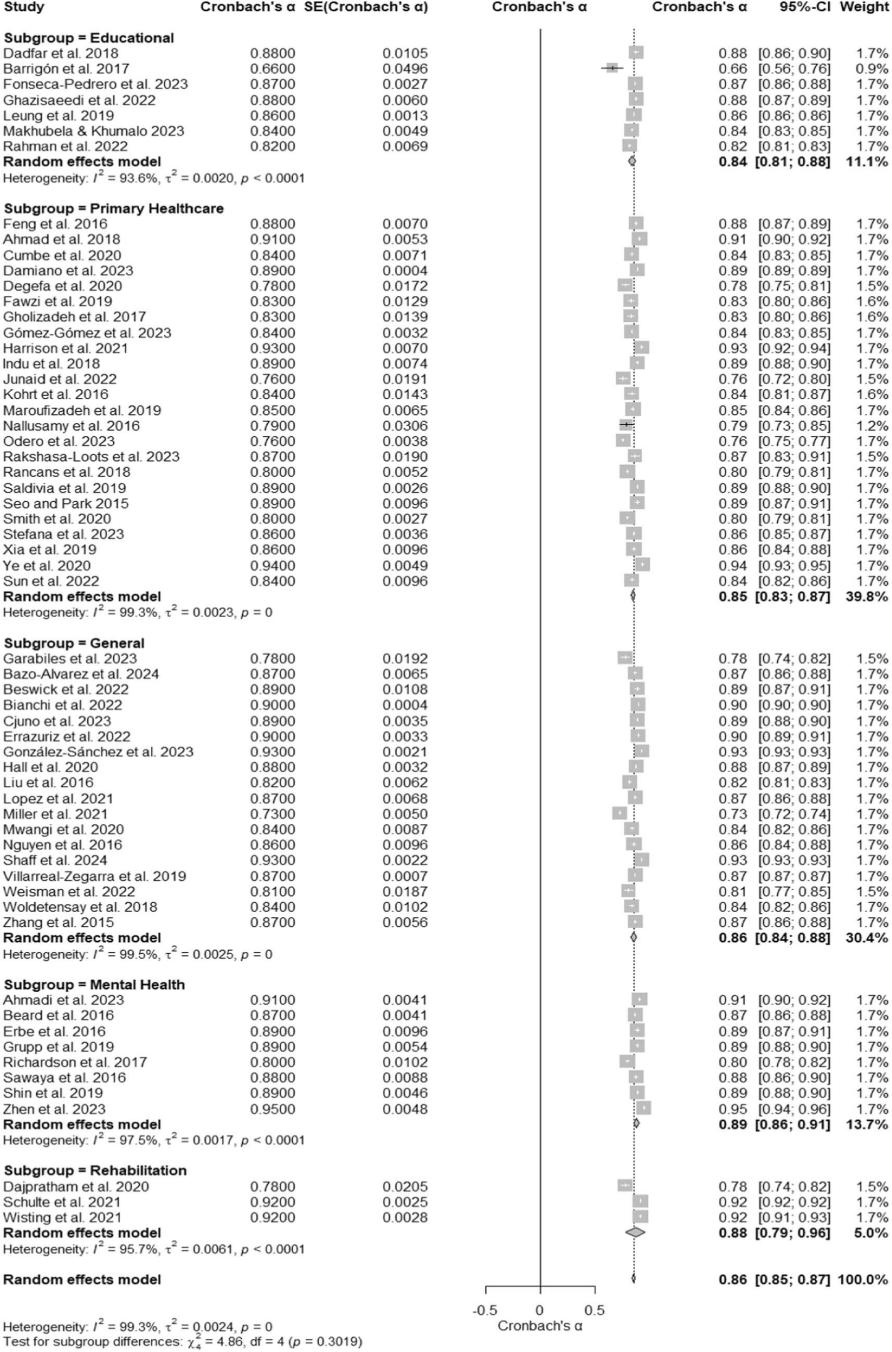
Subgroup Forest Plot of Cronbach’s α for the PHQ- 9 by Setting. This forest plot presents Cronbach’s α reliability estimates for the PHQ- 9 across subgroups based on study settings, including educational, primary healthcare, general population, mental health, and rehabilitation. Individual study estimates, subgroup pooled estimates, and confidence intervals are displayed. Heterogeneity is assessed within and across subgroups

A meta-analysis of 60 studies evaluated the internal consistency reliability of the Patient Health Questionnaire- 9 (PHQ- 9) across different settings. The overall Cronbach’s α was 0.86 (95% CI [0.85, 0.87]), as displayed in the forest plot (Fig. 6), indicating high reliability across diverse contexts. Substantial heterogeneity was observed, with I^2^ = 99.3%, τ^2^ = 0.0024, and a significant test of heterogeneity, Q (59) = 7981.54, p < 0.001 (Table 1). Subgroup analyses revealed Cronbach’s α values ranging from 0.84 to 0.89 across different settings. Educational settings had a Cronbach’s α of 0.84 (95% CI [0.81, 0.88]) with I^2^ = 93.6%, while primary healthcare settings demonstrated a Cronbach’s α of 0.85 (95% CI [0.83, 0.87]) with I^2^ = 99.3%. General settings reported a Cronbach’s α of 0.86 (95% CI [0.84, 0.88]) with I^2^ = 99.5%, and mental health settings showed the highest reliability with a Cronbach’s α of 0.89 (95% CI [0.86, 0.91]) and I^2^ = 97.5%. Rehabilitation settings had a Cronbach’s α of 0.88 (95% CI [0.79, 0.96]) with I^2^ = 95.7%. The test for subgroup differences was not significant, Q (4) = 4.86, p = 0.3019, indicating no significant variability in reliability across settings.

### PHQ- 9 reliability by mode of administration

**Fig. 7:**
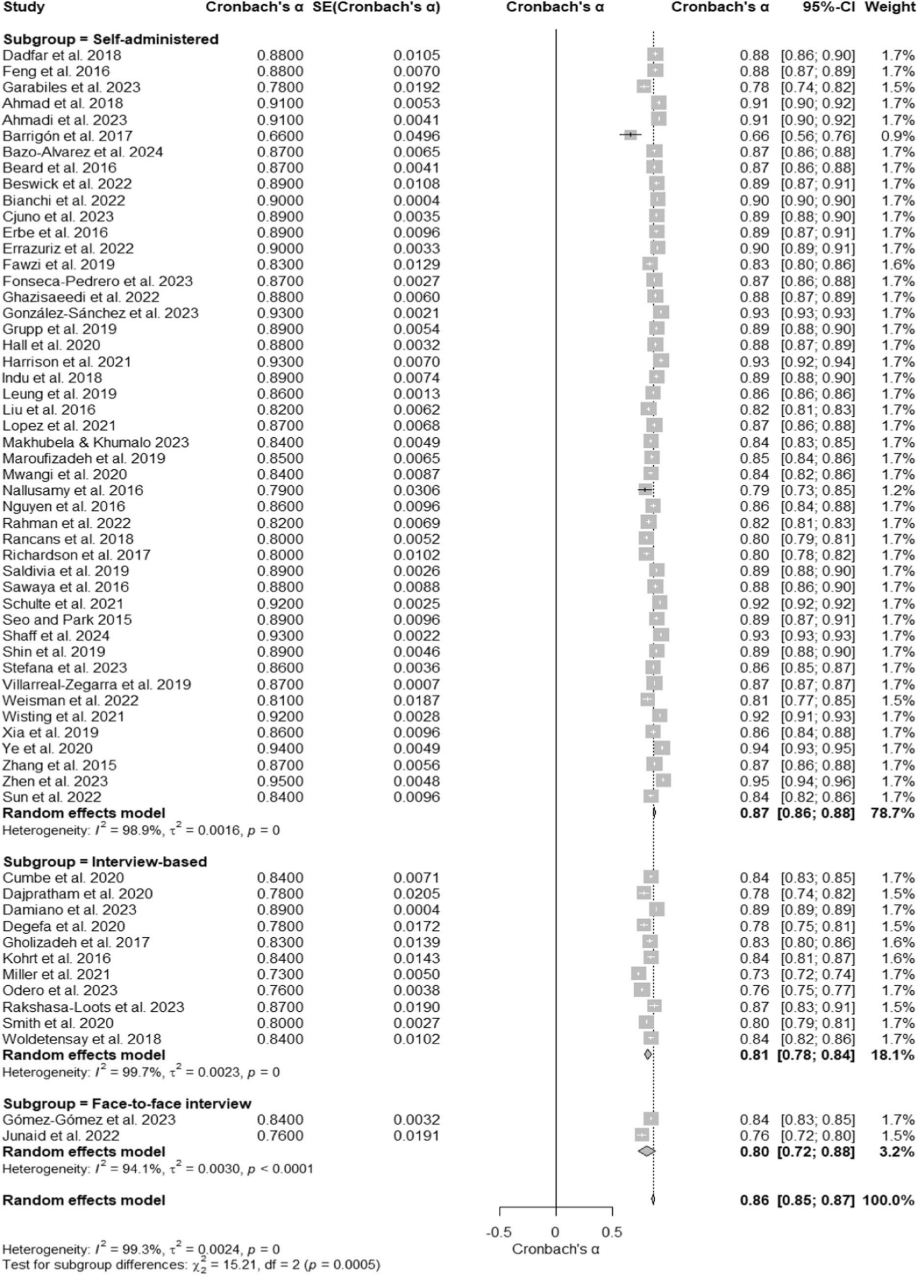
Subgroup Forest Plot of Cronbach’s α for the PHQ- 9 by Mode of Administration. This forest plot displays Cronbach’s α reliability estimates for the PHQ- 9 across subgroups based on the mode of administration, including self-administered, interview-based, and face-to-face formats. Individual study estimates, subgroup pooled estimates, and their respective confidence intervals are shown. Heterogeneity is assessed within and across subgroups

A meta-analysis of 60 studies evaluated the internal consistency reliability of the Patient Health Questionnaire- 9 (PHQ- 9) across different modes of administration. The overall Cronbach’s α was 0.86 (95% CI [0.85, 0.87]), as displayed in the forest plot (Fig. 7), reflecting high reliability across diverse administration methods. Substantial heterogeneity was observed, with I^2^ = 99.3%, τ^2^ = 0.0024, and a significant test of heterogeneity, Q (59) = 7981.54, p < 0. 001. Subgroup analyses showed variations in Cronbach’s α across modes of administration. Self-administered questionnaires had the highest reliability, with a Cronbach’s α of 0.87 (95% CI [0.86, 0.88]), and heterogeneity at I^2^ = 98.9%. Interview-based modes demonstrated a Cronbach’s α of 0.81 (95% CI [0.79, 0.84]) with I^2^ = 99.7%. Face-to-face interviews reported the lowest reliability, with a Cronbach’s α of 0.80 (95% CI [0.72, 0.88]), and heterogeneity at I^2^ = 94.1% (Table 1). The test for subgroup differences was significant, Q (2) = 15.21, p = 0.0005, indicating that the mode of administration affects the reliability of the PHQ- 9.

### PHQ- 9 reliability across regions

**Fig. 8:**
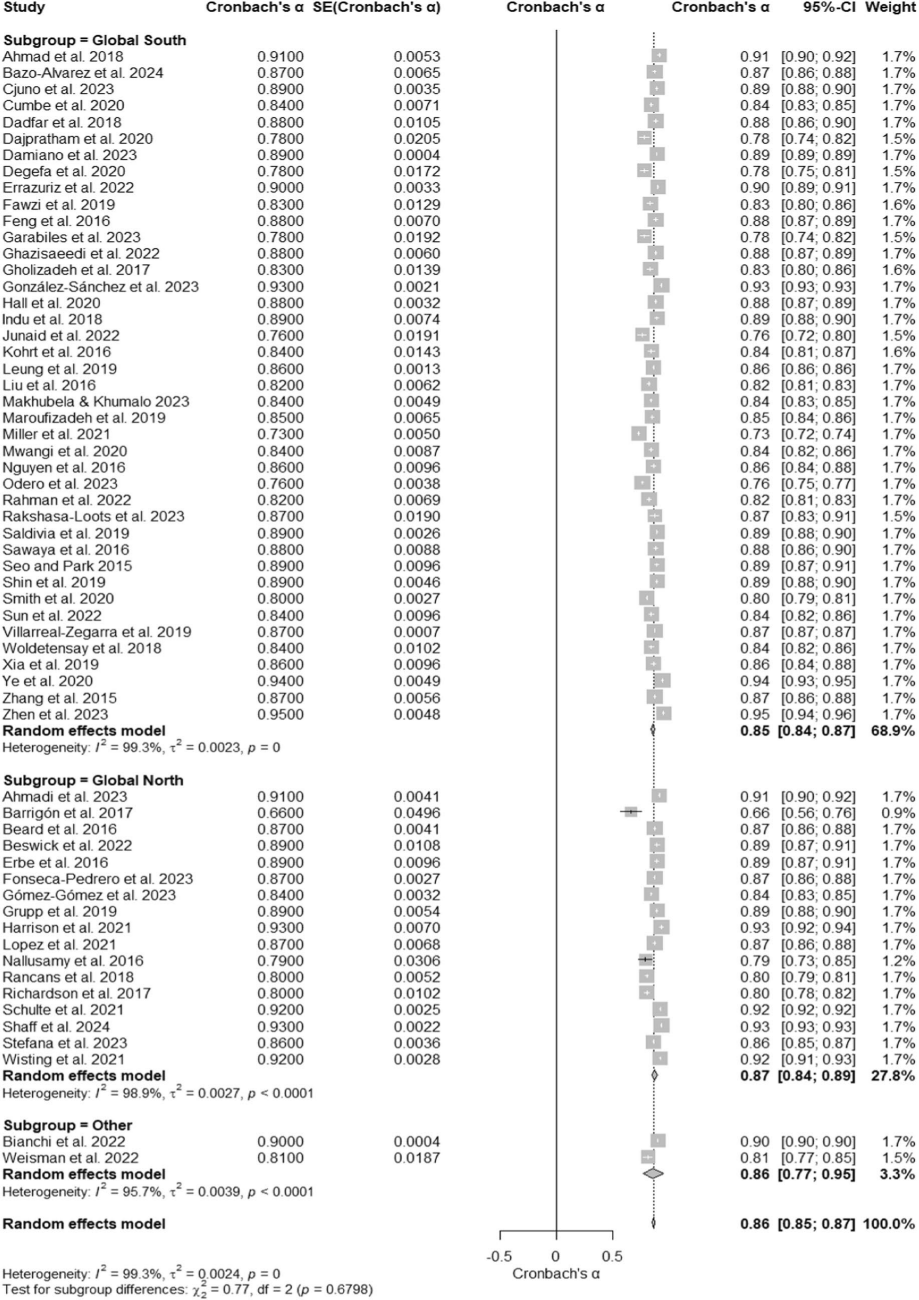
Subgroup Forest Plot of Cronbach’s α for the PHQ- 9 by Region. This forest plot presents Cronbach’s α reliability estimates for the PHQ- 9 across subgroups based on region, including Global South, Global North, and other regions. Individual study estimates, subgroup pooled estimates, and their respective confidence intervals are displayed. Heterogeneity is assessed within and across subgroups

A meta-analysis of 60 studies evaluated the internal consistency reliability of the Patient Health Questionnaire- 9 (PHQ- 9) across different regions. The overall Cronbach’s α was 0.86 (95% CI [0.85, 0.87]), as displayed in the forest plot (Fig. 8), indicating high reliability across diverse geographical contexts. Substantial heterogeneity was observed, with I^2^ = 99.3%, τ^2^ = 0.0024, and a significant test of heterogeneity, Q (59) = 7981.54, p < 0.001 (Table 1). Subgroup analyses revealed that studies conducted in the Global South reported a Cronbach’s α of 0.85 (95% CI [0.84, 0.87]) with I^2^ = 99.3%. Studies from the Global North demonstrated slightly higher reliability, with a Cronbach’s α of 0.87 (95% CI [0.84, 0.89]) and I^2^ = 98.9%. The “Other” subgroup, which included studies not specifically categorized as Global North or South, had a Cronbach’s α of 0.86 (95% CI [0.77, 0.95]) with I^2^ = 95.7%. The test for subgroup differences was not significant, Q (2) = 0.77, p = 0.6798, suggesting that regional differences do not significantly impact the reliability of the PHQ- 9.

### PHQ- 9 reliability by sample size

**Fig. 9:**
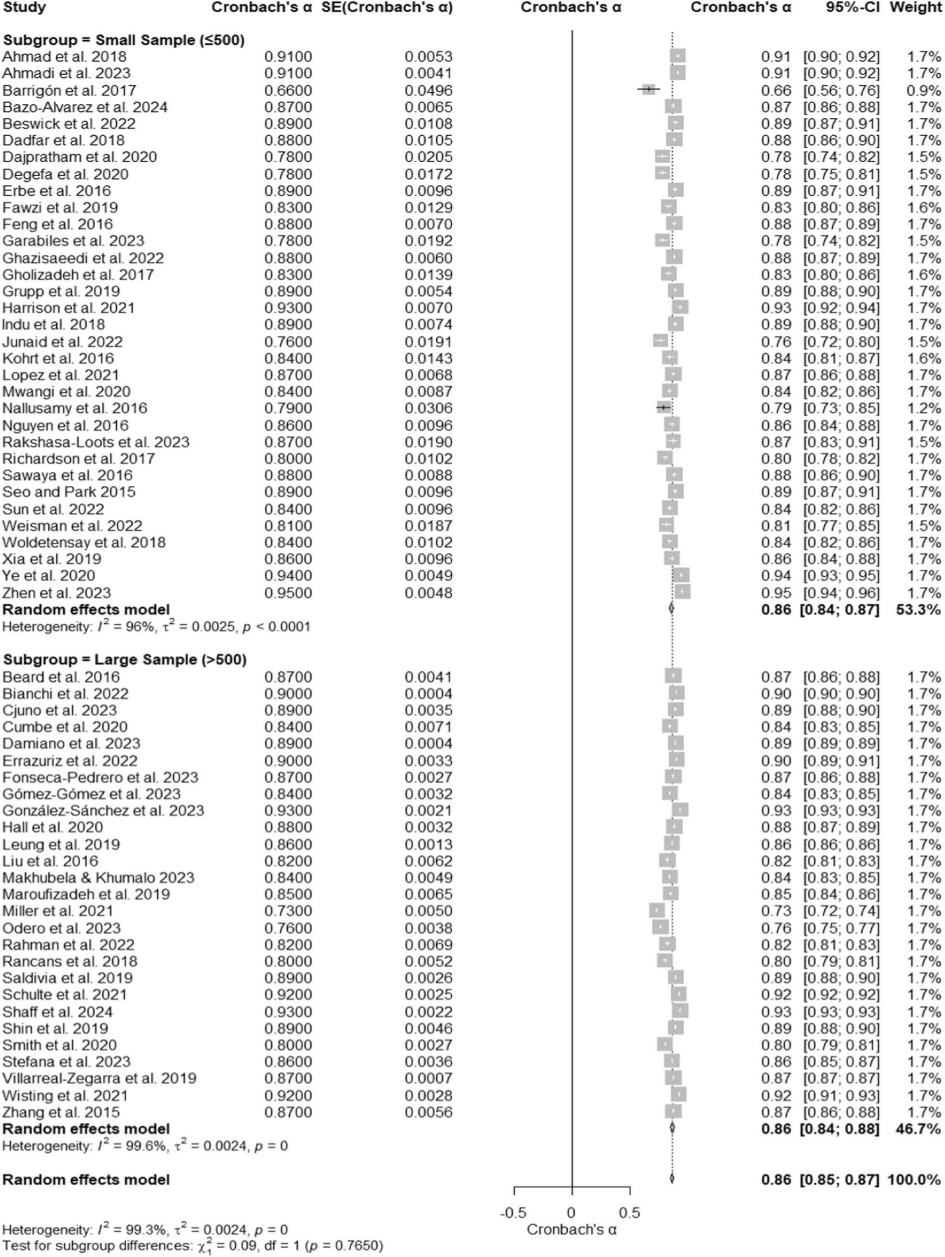
Subgroup Forest Plot of Cronbach’s α for the PHQ- 9 by Sample Size. This forest plot presents Cronbach’s α reliability estimates for the PHQ- 9, stratified by sample size subgroups (small samples ≤ 500 and large samples > 500). Individual study estimates, subgroup pooled estimates, and their respective confidence intervals are shown. Heterogeneity is assessed within and across subgroups

A meta-analysis of 60 studies evaluated the internal consistency reliability of the Patient Health Questionnaire- 9 (PHQ- 9) across different sample sizes. The overall Cronbach’s α was 0.86 (95% CI [0.85, 0.87]), as displayed in the forest plot (Fig. 9), indicating high reliability regardless of sample size. Substantial heterogeneity was observed, with I^2^ = 99.3%, τ^2^ = 0.0024, and a significant test of heterogeneity, Q (59) = 7981.54, p < 0.001. Subgroup analyses revealed similar reliability estimates for studies with small samples (≤ 500 participants) and large samples (> 500 participants). Studies with small samples reported a Cronbach’s α of 0.86 (95% CI [0.84, 0.87]), with heterogeneity at I^2^ = 96.0% (Table 1). Studies with large samples demonstrated a Cronbach’s α of 0.86 (95% CI [0.84, 0.88]), with heterogeneity at I^2^ = 99.6%. The test for subgroup differences was not significant, Q (1) = 0.09, p = 0.7650, suggesting that sample size does not significantly affect the reliability of the PHQ- 9.

## Discussion

Our meta-analysis supports the high reliability of the PHQ- 9 as a depression screening tool, consistent with prior research. The consistency of its reliability across different languages and cultural adaptations supports its global applicability [5, 53, 54]. The PHQ- 9 has been found to have good reliability and validity in various populations, including psychiatric hospital sample settings [13, 14] and the general population in different countries [3, 6, 37]. It has been widely used and recommended for evaluating depressive symptoms in clinical trials, research, and primary care settings [11, 36]. The internal consistency reliability of the PHQ- 9 has been consistently high, indicating its stability and accuracy in measuring depression severity. The PHQ- 9 has shown strong evidence of validity and reliability, establishing it as a useful instrument for screening depression.

The high test–retest reliability observed in various formats and settings further endorses the PHQ- 9’s stability over time [12, 15, 41]. The adaptation of the PHQ- 9 for different cultural groups and its validation across various demographics illustrate the instrument’s flexibility and the potential for its broader application in global mental health [9, 50]. Our meta-analysis affirms the strong reliability of the PHQ- 9 across various settings and populations, consistent with previous validation studies that highlight its robust psychometric properties [3, 11]. Notably, the tool’s adaptability to different languages and cultural contexts without significant loss of reliability supports its use in global mental health assessments, reinforcing findings from studies in diverse cultural settings [53, 54].

The meta-analysis revealed high-reliability coefficients for the PHQ- 9 across various settings and populations, echoing the robust psychometric properties initially reported by Kroenke, Spitzer, and Williams [3] and later supported by the findings of Villarreal-Zegarra et al. [50] and Bianchi et al. [5]. The consistency of these results across diverse linguistic and cultural adaptations, as seen in the works of Bazo-Alvarez et al. [19] and Dadfar et al. [9], illustrates the PHQ- 9’s effectiveness in capturing the essence of depressive symptoms across different cultural contexts. This is particularly relevant given the identified variability in the factorial structure of the PHQ- 9 among different populations, as highlighted by Blake et al. [7] and Tibubos et al. [8]. Such variability underscores the importance of considering cultural details in the expression and understanding of depression, which may not be fully encapsulated by the original PHQ- 9 framework.

The findings of substantial heterogeneity in reliability scores based on the mode of administration and the setting, as observed in our meta-analysis, highlight the complexity of deploying a universal screening tool like the PHQ- 9 in varied clinical and non-clinical environments. This observation prompts a deeper exploration into how specific cultural and administrative contexts influence the reliability and accuracy of depression assessments, echoing the concerns [55, 56].

### Limitations and future research

This study has several limitations that warrant discussion. First, it focuses solely on the reliability of the PHQ- 9 and does not evaluate its validity. While validity is a critical psychometric property, our meta-analysis was designed to assess the consistency of PHQ- 9 scores across diverse populations and settings. Evaluating validity would require a different methodological approach, including assessments of criterion, construct, and content validity. Future research should investigate the validity of the PHQ- 9, particularly its measurement invariance across cultural and linguistic adaptations.

Second, the substantial heterogeneity observed in this meta-analysis (I^2^ = 99.3%) underscores variability in reliability across studies. While subgroup analyses helped identify some factors contributing to this heterogeneity, unmeasured variables such as healthcare infrastructure, training of administrators, and cultural differences may also play a role. Future research should further explore these contextual factors to enhance the PHQ- 9’s applicability in diverse settings.

Additionally, this study does not evaluate the PHQ- 9’s impact on clinical decision-making or its role in prescribing practices. While the PHQ- 9 is widely used to inform clinical decisions, this study focuses solely on its measurement reliability. Future research should explore how PHQ- 9 scores influence clinical decision-making and their implications for treatment strategies in diverse healthcare settings.

Lastly, this meta-analysis included only peer-reviewed, English-language studies, which may introduce publication bias and limit generalizability to non-English-speaking regions. Expanding the inclusion criteria to include grey literature and studies published in other languages could provide a more comprehensive understanding of the PHQ- 9’s reliability. Despite these limitations, the study provides robust evidence for the PHQ- 9’s reliability, supporting its continued use as a global tool for depression screening and monitoring. Future research addressing these gaps will further optimize its utility and contribute to improving mental health outcomes worldwide.

### Implications for practice and research

The findings of this meta-analysis have significant implications for both practice and research in global mental health. The PHQ- 9, with its demonstrated high reliability across diverse populations and settings, remains a robust tool for depression screening and monitoring. Its adaptability to different languages, cultural contexts, and administration modes underscores its value in resource-limited settings and cross-cultural studies. However, the observed heterogeneity in reliability emphasizes the need for ongoing validation and adaptation of the PHQ- 9 to ensure it captures culturally specific expressions of depression accurately.

For clinical practice, the PHQ- 9 offers a reliable measure for early detection and intervention, supporting healthcare providers in tailoring treatments to individual patient needs. In research, the significant variability in reliability across languages, regions, and modes of administration highlights the importance of accounting for contextual factors when using the PHQ- 9 in studies. Future research should prioritize evaluating the PHQ- 9’s validity, including its measurement invariance across cultures and its applicability in non-clinical populations. Additionally, further exploration of its performance in digital formats could enhance its utility in telemedicine and remote care. By addressing these areas, the PHQ- 9’s utility can be further optimized, contributing to improved mental health outcomes and reducing the global burden of depression.

## Conclusion

Our meta-analysis reinforces the PHQ- 9’s position as a highly reliable and versatile instrument for depression screening and monitoring, with proven efficacy across a wide range of global contexts. Also, it confirms that the PHQ- 9 is a highly reliable tool for assessing depression, with its utility extending across numerous languages, cultures, and administration modes. Despite observed heterogeneity in some areas, these findings reinforce the PHQ- 9’s value in clinical and research settings worldwide. By continuing to explore its applications, limitations, and potential adaptations, researchers and clinicians can further enhance its utility and impact in addressing the global burden of depression.

## Permission to reproduce material from other sources

This article has not used material from other sources requiring permission for reproduction. All content presented is original and developed by the authors or falls under open-access licensing, which does not require explicit permission for reproduction in academic publications.

## Data Availability

The data supporting this study’s findings are available from the corresponding author upon reasonable request.
